# Unlocking New Horizons: Antibody–Drug Conjugates in Small Cell Lung Cancer

**DOI:** 10.3390/biology14121677

**Published:** 2025-11-26

**Authors:** Jiayu Liu, Yan Cui, Dongying Liao, Mingwei Sima, Moxuan Han, Wanhao Li, Yan Bi, Donghui Yue

**Affiliations:** 1 School of Basic Medicine, Changchun University of Chinese Medicine, Changchun 130117, China; 13611371417@139.com (J.L.); cycczyydx@163.com (Y.C.); 2 School of Chinese Classics, Beijing University of Chinese Medicine, Beijing 100029, China; liaodongying0106@163.com; 3 School of Traditional Chinese Medicine, Changchun University of Chinese Medicine, Changchun 130117, China; smmwyjs@163.com (M.S.); 23102570153@stu.ccucm.edu.cn (M.H.); li8678478@163.com (W.L.); biyan407@126.com (Y.B.)

**Keywords:** antibody–drug conjugate, small cell lung cancer, tumour therapy, research progress

## Abstract

Antibody-drug conjugates (ADCs), an emerging class of antitumor agents, have revolutionised the therapeutic landscape in oncology by integrating the cytotoxic efficacy of chemotherapy with the precision of targeted therapy. These agents have also demonstrated considerable potential in the treatment of small cell lung cancer (SCLC). Numerous clinical trials evaluating ADCs in SCLC are currently underway. Although encouraging outcomes have been observed, several challenges persist, including drug resistance, treatment-related toxicities, and the identification of suitable biomarkers for patient selection. A comprehensive understanding of the mechanisms underlying ADCs, along with a systematic review of their clinical development in SCLC, may provide valuable insights and strategies to address these issues. In this review, we elucidate the structure and mechanisms of action of ADCs, summarise key clinical trials in SCLC, discuss existing challenges, and suggest future directions to facilitate the advancement and clinical application of ADC-based therapies for SCLC.

## Simple Summary

The advent of antibody–drug conjugates (ADCs) mark a transformative advance in the treatment of small cell lung cancer (SCLC), a malignancy noted for its aggressive course and limited therapeutic options. Whereas earlier work established the roles of chemotherapy and immunotherapy, this review highlights a new paradigm: ADCs combine the selectivity of targeted therapy with the potent cytotoxicity of conventional chemotherapy, enabling the targeted delivery of highly active payloads to tumour cells. This strategy has shown promising efficacy against targets such as DLL3, B7-H3, TROP-2 and SEZ6, which are highly expressed in SCLC. Significant challenges nevertheless remain, including treatment-related toxicities and the emergence of drug resistance. A clearer understanding of ADC mechanisms, together with a systematic appraisal of their clinical development, reveals important opportunities. By optimising ADC design, identifying predictive biomarkers, and devising rational combination strategies, researchers can pursue more effective and durable therapies for patients with SCLC, thereby improving survival in this hard-to-treat disease.

## 1. Introduction

Lung malignancies represent the foremost cause of cancer-related mortality worldwide [1]. Around 2.48 million new cases of lung cancer were diagnosed in 2022, making up 12.4% of all cancer cases, and the disease was responsible for over 1.8 million deaths, or 18.7% of all cancer deaths, according to global cancer statistics [2]. The above statistics highlight the serious risk that lung cancer presents to public health around the world [3]. Based on the histopathological type, lung cancer can be divided into SCLC and non-small cell lung cancer (NSCLC) [4]. SCLC, which accounts for approximately 15% of all lung tumours [5], is characterised by rapid growth, a high degree of vascularization, genomic instability, high malignancy, and a strong predilection for early metastasis, making it a difficult area for clinical treatment and a poor prognosis [6]. Chemotherapy has been the standard treatment option for extensive disease (ED)-SCLC since the 1980s; however, the mOS (median overall survival) of patients has never reached one year with either etoposide plus cisplatin (EP), etoposide plus carboplatin (EC) or other chemotherapy regimens [7,8]. In recent years, immune checkpoint inhibitors (ICIs) have extended survival in SCLC patients, yet treatment responses remain modest and resistance to immunotherapy is inevitable [9]. Consequently, numerous unmet clinical needs persist in the field of SCLC. ADCs have gradually made significant breakthroughs in the treatment of breast cancer, gastric cancer and other solid tumours, since the approval of the first ADC called mylotarg by the US Food and Drug Administration (FDA) in 2000 [10]. According to statistics, global enthusiasm for ADC research and development continues to rise, with 16 ADCs currently approved and over 800 in development [11]. ADCs also have great potential in the treatment of SCLC.

The development of ADCs was based on Paul Ehrlich’s conception of the “magic bullet” theory and a concept of selective delivery, which inspired generations of scientists to devise powerful molecular cancer therapeutics [12]. ADCs are a class of biological drugs consisting of monoclonal antibodies and potent cytotoxic small molecule drugs coupled by bioactive linkers [13]. After ADC enters the blood circulation, its antibody part can recognise and bind to the specific antigen on the surface of tumour cells, forming an ADC–antigen complex, which enters the tumour cells through endocytosis and releases cytotoxic drugs after being degraded by lysosomes to destroy DNA or prevent tumour cells from dividing, thus playing the role of killing tumour cells [14]. Therefore, ADC combines the characteristics of highly specific and targeted monoclonal antibody drugs with the high efficiency of cytotoxic drugs in removing cancer cells [15], which can synergistically utilise the respective advantages of antibody drugs and chemical drugs to improve efficacy and reduce systemic toxicity [16].

**Figure 1:**
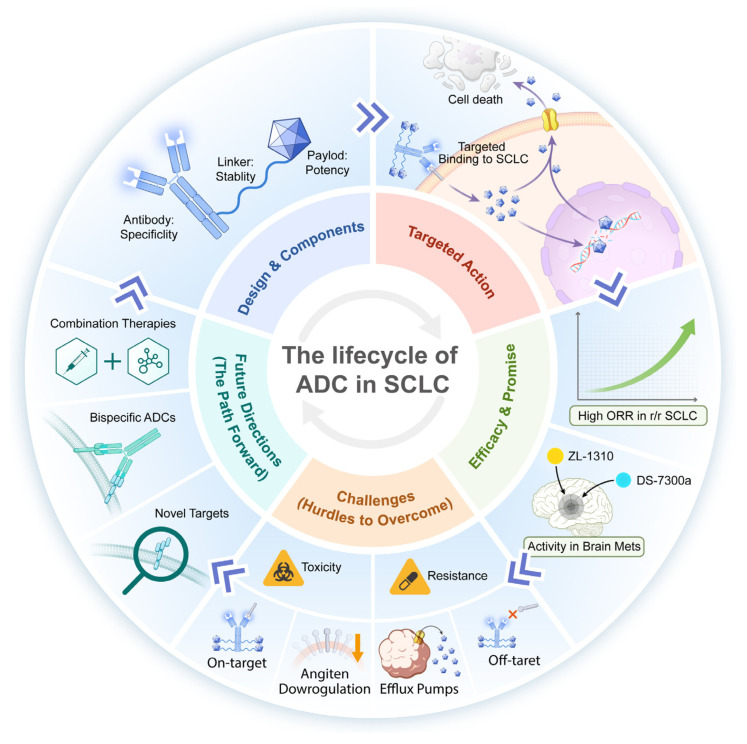
The therapeutic landscape, mechanisms of action, and primary challenges of ADCs in SCLC. ADCs constitute a promising therapeutic class for SCLC, designed to deliver potent cytotoxins selectively to tumour cells. The mechanism begins with the antibody component conferring specificity by binding to surface antigens on SCLC cells. After internalisation, the ADC is processed in lysosomes, where the linker, which is crucial for systemic stability, is cleaved to release the payload; this payload then exerts its potency by inducing cell death. This targeted lifecycle of ADCs in SCLC underlies their notable efficiency and promise, including reports of high objective response rates (ORR) in relapsed/refractory (r/r) SCLC patients and demonstrated activity in brain metastases (Mets). However, clinical application faces significant challenges. Toxicity remains dose limiting, as observed with specific ADCs such as ZL-1310 and DS-7300a. In addition, the emergence of resistance curtails long-term efficacy. Key resistance mechanisms included on-target effects, for example, antigen downregulation that reduces ADC binding, and off-target mechanisms, notably upregulation of efflux pumps that actively expel the payload from tumour cells. Future directions to overcome these hurdles focused on next-generation strategies, including bispecific ADCs to enhance targeting, exploration of novel targets to circumvent resistance, and rational combination therapies to improve overall antitumour activity.

Currently, ADC technology is widely employed in the field of oncology and has been utilised in clinical practice for the treatment of various solid tumours, including breast cancer [17], gastric cancer [18], and prostate cancer [19]. Clinical studies targeting SCLC are also extensively underway, primarily involving the following targets: DLL-3, CD56, TROP-2, and B7-H3. In recent years, ADCs against different targets have proved to be effective in clinical studies in SCLC. In this article, we systematically review the principles of ADCs, recent research progress, challenges, and prospects in the field of SCLC. This article provides a review of clinical studies of ADCs in SCLC (Figure 1).

## 2. Fundamentals and Mechanism of ADC Drugs

### 2.1. The Fundamentals of ADCs

Typically, ADCs consist of three parts: the payload, the antibody, and the linker. [20]. Antibody is usually a specific monoclonal antibody that recognises and binds to a particular antigen on the surface of cancer cells. Payload a cytotoxic small molecule drug that kills tumour cells or stops them from proliferating. A linker is a chemical link that connects the antibody to the payload, which can be cleavable or non-cleavable and determines how the payload is released once it reaches the cancer cell. ADCs represent cutting-edge biopharmaceutical technology that conjugates highly specific monoclonal antibodies to potent cytotoxic drugs via chemical linkers [21]. The specific modes of operation for the three are as follows:

#### 2.1.1. Payload

The payload, also termed the projectile or cytotoxic drug, has been demonstrated to possess antitumour activity. Presently, the cytotoxic drugs or payloads commonly employed in clinical practice are predominantly microtubule inhibitors, with a minority being DNA-damaging agents. The latter category encompasses cytotoxic drugs capable of inducing various types of damage, including DNA double-strand breaks, DNA alkylation, and DNA cross-linking [22]. The development of an ideal cytotoxic payload for antibody–drug conjugates must satisfy three core criteria. First, it must be highly stable in systemic circulation and display low intrinsic toxicity to minimise off-target effects. Second, once internalised by target tumour cells, it must release its active form efficiently to produce potent cytotoxicity. Third, it should have low immunogenicity, a small molecular weight, and a long half-life. This combination enables precise tumour-cell killing and contributes to a wider therapeutic index.

#### 2.1.2. Antibody

Antibodies serve as the primary component, enabling cytotoxic drugs to bind to specifically expressed or highly expressed tumour surface targets through antibody action, thereby targeting tumour cells. Consequently, antibody-mediated cytotoxicity achieves the direct suppression or elimination of tumour cells. An ideal antibody should possess multiple characteristics: high specificity, high affinity, long half-life, and low immunogenicity [23]. In the selection of antibodies, mouse-derived antibodies were frequently chosen in the early stages. However, mouse-derived antibodies induce an immunogenic response in the body, diminishing the efficacy of ADCs and increasing cytotoxicity [24]. Today, humanised antibodies are prioritised in clinical practice to minimise immunogenicity. These primarily derive from immunoglobulin IgG, comprising four subclasses: IgG1, IgG2, IgG3, and IgG4. IgG1 monoclonal antibodies are predominantly used as the foundational structure for ADCs due to their stability and straightforward production methods [25]. Additionally, IgG1 provides robust antibody-dependent cellular cytotoxicity and complement-dependent cytotoxicity.

#### 2.1.3. Linker

**Figure 2:**
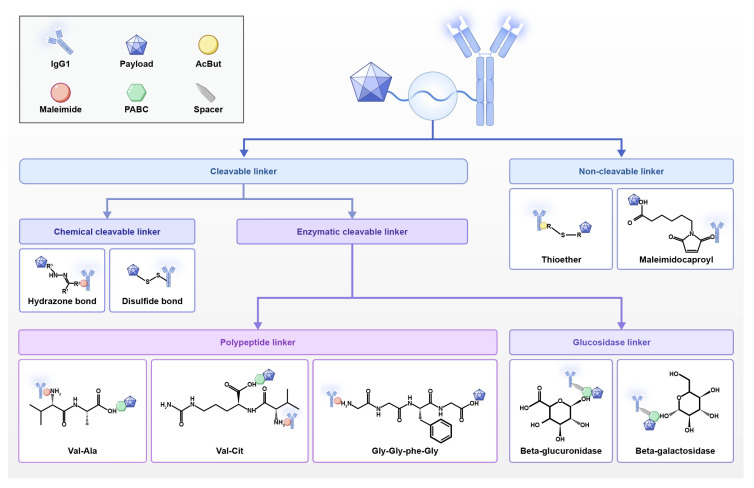
Linkers chemistry for ADCs. This schematic illustrates the core structural components of ADCs. linkers, as key components for payload release, are primarily categorised into two types: Cleavable linkers: designed to release the payload within specific intracellular environments. Chemically cleavable linkers: susceptible to endosomal/lysosomal-specific environments, incorporating acid-labile hydrazine bonds or redox-sensitive disulphide bonds. Enzymatically cleavable linkers: lysosomal protease substrates, such as the dipeptide motifs Val-Cit and Val-Ala, or the tetrapeptide motif Gly-Gly-Phe-Gly. Additionally includes linkers cleaved by lysosomal enzymes such as β-glucuronidase and β-galactosidase. Non-cleavable linkers, such as thioethers or maleimide hexanoyl, relying on complete degradation of the antibody fragment within lysosomes to release the active payload. Spacer groups (e.g., PABC, AcBut) link the linker to the payload, typically employed to optimise stability, solubility, and payload-release efficiency upon linker cleavage. Maleimide groups are commonly used chemical linkers connecting the linker to the antibody.

The linker is an indispensable component of ADCs, playing a crucial role. As the core functional component of ADCs, it covalently links cytotoxic payloads to monoclonal antibodies, thereby precisely regulating targeted drug delivery: on the one hand, a suitable linker must maintain high stability in the bloodstream and resist premature cleavage to ensure the structural integrity of the ADC. On the other hand, it must ensure specific cleavage within tumour cells to release the cytotoxic drug [26]. Sufficient circulatory stability effectively prevents premature drug release in plasma, reducing its toxicity to healthy cells and thereby enhancing its tumour-killing efficacy [27]. Based on their cleavage characteristics, linkers are categorised into cleavable and non-cleavable types (Figure 2). Cleavable linkers exhibit lower stability, cleaving within the tumour microenvironment to release the drug. This may induce a ‘bystander effect’, killing neighbouring cancer cells and broadening the antitumour response [28]. Non-cleavable linkers, conversely, form irreversible chemical bonds, demonstrating greater stability in circulation. Drug release primarily relies on lysosomal degradation following antibody internalisation. This characteristic has led to their widespread adoption in clinical applications.

### 2.2. The Mechanism of Action of ADCs

**Figure 3:**
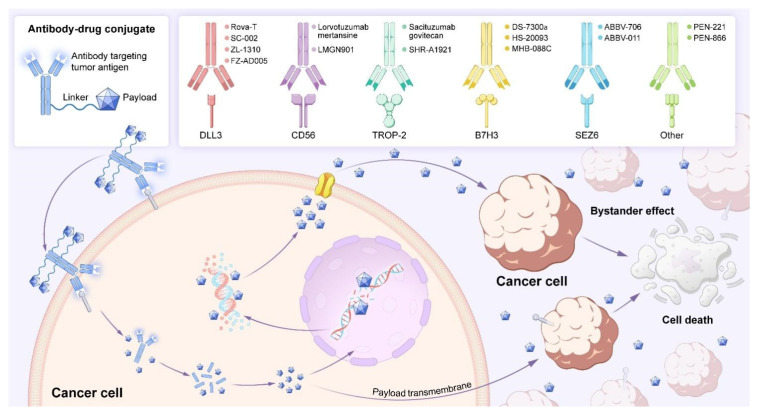
Mechanism of action and representative targeted therapeutics of ADCs. This schematic illustrates the mechanism by which ADCs mediate targeted killing of cancer cells. An ADC comprises a monoclonal antibody chemically linked via a spacer to a potent cytotoxic payload. The antibody moiety specifically binds tumour-associated antigens on cancer cell surfaces (e.g., DLL3, CD56, TROP-2, B7-H3, SEZ6). Following binding, the ADC-antigen complex is internalised via endocytosis. Subsequently, the ADC is transported to the lysosomal compartment, where the linker is cleaved, releasing the active cytotoxic payload (e.g., PBD dimer, SN-38, DMx) into the cytoplasm. This payload then exerts its mechanism of action (e.g., inducing DNA damage or disrupting microtubule assembly), ultimately leading to programmed cell death. Representative ADCs in clinical development for each target are shown in the upper right of the figure.

ADCs exert antitumour effects via a multistep mechanism [29] (Figure 3). As macromolecules, ADCs preferentially extravasate through tumour leaky vasculature and bind target antigens on cancer cell surfaces. Upon binding, the ADC–antigen complex is internalised by clathrin-mediated endocytosis and trafficked to endosomes and lysosomes. Within these compartments, the linker is cleaved under specific conditions—such as low pH, enzymatic activity, or a reductive environment—and the cytotoxic payload is released [30]. Linker design is critical because it governs both the timing and efficiency of drug release. The released payload induces tumour cell death by disrupting essential cellular processes, including microtubule assembly or DNA integrity. Furthermore, certain lipophilic payloads diffuse across cell membranes and kill adjacent antigen-negative tumour cells; this phenomenon is known as the “bystander effect.” This effect is particularly advantageous in heterogeneous tumours but can also contribute to on-target off-tumour toxicity if the payload damages neighbouring normal cells [31]. Thus, although ADCs represent a targeted therapeutic strategy that combines precise antibody guidance with potent cytotoxicity, their efficacy and safety rely on the coordinated function of all three components: antibody, linker, and payload. Optimisation of each element is essential to maximise antitumour activity while minimising adverse effects.

## 3. Targeting SCLC: Potential Targets and Advanced ADCs

Numerous targets for ADCs have been identified in SCLC, as detailed below. Table 1 summarises the primary targets, corresponding drugs, and related information for the drugs discussed in this paper. Table 2 summarises the relevant clinical trials currently underway.

### 3.1. DLL3-Targeting ADCs

Existing studies indicate that the Notch signalling pathway plays a crucial role in the pathogenesis and progression of SCLC through the regulation of key cellular processes including proliferation, differentiation, and apoptosis [32]. Preclinical studies demonstrate widespread expression of DLL-3 in tumour neuroendocrine cells, with approximately 80% of SCLC patients harbouring this receptor [33]. DLL3 is a transmembrane protein belonging to the Notch ligand family [34]. It inhibits Notch activation during embryonic development and neuroendocrine cell differentiation by suppressing the Notch signalling pathway, thereby promoting tumourigenesis and tumour progression [35]. Currently, the following ADC drugs targeting DLL3 are available.

**Table 1:** Summary of ADCs and SMDCs in Clinical Development for SCLC.

Target	Agent Name	Linker/Payload Mechanism	Clinical Phase	Efficacy Outcomes (ORR/PFS)	Key Toxicities	Status/Outcome	References
DLL3	Rovalpituzumab Tesirine (Rova-T)	Not specified/Tesirine (DNA-damaging agent)	I–III	Phase II: ORR 12.4% (all), mPFS ~4.6 mo (Phase I)	Fatigue, pleural effusion, thrombocytopenia, skin reactions, photosensitivity	Clinical development terminated. Failed to show survival benefit vs. standard care.	[36,37,38,39,40,41,42]
DLL3	SC-002	Different chemical linker vs. Rova-T/Not specified	Early-phase	ORR 14% (all), 11.8% (DLL3-positive)	Serous cavity effusion, dyspnea, thrombocytopenia	Study discontinued. Limited efficacy and significant toxicity.	[43]
DLL3	ZL-1310	Cleavable linker/Topoisomerase I inhibitor	I (Ongoing, NCT06179069)	ORR in brain metastases: 80%; DCR: 100%	Anaemia, neutropenia, thrombocytopenia	Active, promising. Manageable safety and notable activity, especially in brain metastases.	[44,45]
DLL3	FZ-AD005	Cathepsin-cleavable linker/DXd (Topo I inhibitor)	I (Ongoing, NCT06424665)	Primary endpoints include ORR (data pending)	DLTs, AEs under evaluation	Active, early-stage. First-in-human study assessing safety and preliminary activity.	[46,47]
DLL3	SHR-4849	Not specified/Not specified	I	ORR 73.2% (at ≥2.4 mg/kg); DCR 93.0%; DCR in brain metastases: 100%	Manageable safety profile, well-tolerated	Active, highly promising. High response rates support further clinical development.	[48]
B7-H3	DS-7300a (I-DXd)	Cleavable tetrapeptide linker/I-DXd (Topo I inhibitor)	II	ORR 48.2%; DCR 87.6%; mPFS 4.9 mo; mOS 10.3 mo; intracranial ORR 46.2%	Safety profile deemed manageable	Breakthrough therapy designation. Robust and durable activity in pre-treated ES-SCLC.	[49,50,51,52]
B7-H3	HS-20093	Not specified/Not specified	I/II	ORR: 61.3% (8 mg/kg), 50.0% (10 mg/kg); mPFS: 5.9 mo, 7.3 mo	Neutropenia, leukopenia, thrombocytopenia, anaemia	Active. Promising antitumor activity with haematological toxicities.	[53]
B7-H3	MHB088C (QLC5508)	Not specified/SuperTopoi (potent Topo I inhibitor)	I/II	ORR: 42.9–57.6%; mPFS: 5.5–5.9 mo	Neutropenia, thrombocytopenia, anaemia; mild ILD (1%)	Active, Phase III planned. Encouraging efficacy and acceptable safety.	[54]
SEZ6	ABBV-011	Non-cleavable linker/Calicheamicin	I	ORR 19% (all), 25% (1 mg/kg cohort); mPFS 3.5 mo	Fatigue, nausea, thrombocytopenia, hepatotoxicity	Development discontinued. Significant hepatotoxicity.	[55,56,57]
SEZ6	ABBV-706	Valine-alanine linker/Topoisomerase I inhibitor	I	ORR in 1.8 mg/kg group: 62.1% (Top1i-naïve); ORR in brain metastases: 62.5%	Anaemia, neutropenia	Active, Phase II initiated. Manageable safety and favourable antitumor activity.	[55,58]
CD56	Lorvotuzumab Mertansine (LM)	Not specified/DM1 (microtubule inhibitor)	I/II	ORR 67.1% (combo); mPFS 6.2 mo	Significant TRAEs leading to death (18/94 patients)	Efficacy limited, toxicity high. Requires optimised therapeutic regimens.	[59,60,61,62,63]
TROP-2	Sacituzumab Govitecan (SG)	Cleavable CL2A linker/SN-38 (Topo I inhibitor)	I/II	TROPiCS-03 (2L): ORR 41.9%; mPFS 4.4 mo; mOS 13.6 mo	Neutropenia, diarrhoea; one death from neutropenic sepsis	Clinically meaningful activity. Favourable tolerability, requires Phase III validation.	[64,65,66,67,68,69]
TROP-2	SHR-A1921	Tetrapeptide cleavable linker/Topoisomerase I inhibitor	I	ORR 33.3%; DCR 66.7%; mPFS 3.8 mo	Stomatitis, nausea, vomiting	Active, promising. Encouraging efficacy and manageable safety in heavily pre-treated patients.	[70,71]
SSTR2	PEN-221 (SMDC)	Not specified/DM1 (maytansinoid)	I/II	Disease stabilisation up to 12 weeks	Well-tolerated in early studies	Early clinical activity. Showed preliminary efficacy in SSTR2-positive SCLC.	[72,73,74,75]
HSP90	PEN-866 (SMDC)	Not specified/SN-38 (Topo I inhibitor)	I/II	Antitumor activity in advanced solid tumours	Expected AE profile, well-tolerated	Active. Evaluated in basket trials; entering clinical development in China.	[76,77]

**Table 2:** Current recruiting, active or ongoing clinical trials on different ADCs involved in SCLC treatment.

Trial/NCT Number	Phase	Agent	Mechanism of Action	Eligibility	Intervention	Primary Endpoint(s)	Key Efficacy Outcomes	Toxicity (≥3 AEs)	Current Status
NCT03061812	III	Rova-T	Anti-DLL3 ADC	DLL3-high ES-SCLC	Rova-T 0.3 mg/kg vs. Topotecan 1.5 mg/m^2^ D1–5	Median PFS, OS	PFS: 3.0 mo (Rova-T) vs. 4.3 mo (Topo); OS: 6.3 mo vs. 8.6 mo	64% vs. 54%	Completed
NCT01901653	I	Rova-T	Anti-DLL3 ADC	Relapsed SCLC	Rova-T 0.2 or 0.3 mg/kg Q6W	ORR, AEs	ORR: 12% (0.3 mg/kg)	39% (Gr 3+ AEs)	Completed
NCT03334487	IIIb	Rova-T	Anti-DLL3 ADC	Relapsed/Refractory DLL3+ SCLC	Rova-T 0.3 mg/kg Q6W + Dexamethasone	PFS, OS	PFS: 3.5 mo; OS: 5.6 mo	58% (Gr 3+)	Completed
NCT03086239	I	Rova-T	Anti-DLL3 ADC	Advanced SCLC	Rova-T 0.2/0.3 mg/kg Q6W + Dexamethasone	PFS, OS	ORR: 14%; PFS: 2.7 mo	52% (Gr 3+)	Completed
NCT03033511	III	Rova-T	Anti-DLL3 ADC	ES-SCLC	Rova-T 0.3 mg/kg Q6W vs. Placebo	PFS, AEs	PFS: 3.0 mo vs. 2.8 mo (NS)	61% vs. 45%	Terminated
NCT02819999	--	Rova-T	Anti-DLL3 ADC	SCLC	Rova-T + Cisplatin/Etoposide	AEs	ORR: 40%	70% (Gr 3+)	Terminated
NCT03026166	I/II	Rova-T	Anti-DLL3 ADC	ES-SCLC	Rova-T + Nivo vs. Rova-T + Nivo/Ipi	ORR, DOR, PFS, OS	ORR: 17% (combo)	54% (Gr 3+)	Completed
NCT03000257	I	Rova-T	Anti-DLL3 ADC	SCLC	Rova-T + Bogriamab (BMS-986012)	ORR	ORR: 15%	48% (Gr 3+)	Completed
(Early-phase ref)	I/II	Rova-T	Anti-DLL3 ADC	Recurrent SCLC	Rova-T Monotherapy	ORR, OS	ORR: 10–15%; OS: ~6 mo	~50% (Gr 3+)	Development Terminated
NCT02500914	I	SC-002	Anti-DLL3 ADC	Refractory SCLC/LCNEC	SC-002 Monotherapy	ORR, Safety	ORR: 0% (early stop)	45% (Gr 3+)	Discontinued
NCT06179069	I	ZL-1310	Anti-DLL3 ADC	r/r ES-SCLC	ZL-1310 Monotherapy	Safety, Efficacy	Pending	Pending	Recruiting
NCT06424665	I	FZ-AD005	Anti-DLL3 ADC	Advanced Solid Tumours	FZ-AD005 (FIH)	DLTs, MTD, AEs	Pending	Pending	Recruiting
NCT06443489	I	SHR-4849	Anti-DLL3 ADC	Advanced Solid Tumours	SHR-4849 Monotherapy	Safety, Tolerability	Pending	Pending	Recruiting
NCT06613009	I	IBJ3009	Anti-DLL3 ADC	SCLC	IBJ3009 Monotherapy	Safety, Tolerability	Pending	Pending	Recruiting
B7-H3									
NCT05280470	II	Ifinatamab Deruxtecan (I-DXd)	Anti-B7-H3 ADC	Pre-treated ES-SCLC	I-DXd 12 mg/kg Q3W	ORR	ORR: 52% (interim)	35% (Gr 3+)	Active, not recruiting
NCT06203210	II/III	Ifinatamab Deruxtecan (I-DXd)	Anti-B7-H3 ADC	Relapsed SCLC	I-DXd vs. Topotecan/Amrubicin	OS, PFS	Pending	Pending	Recruiting
NCT05276609/NCT06498479	I	HS-20093	Anti-B7-H3 ADC	Advanced Solid Tumours	HS-20093 Monotherapy	Safety, Tolerability	Pending	Pending	Recruiting
NCT05914116	I/IIa	HS-20093	Anti-B7-H3 ADC	SCLC	HS-20093 + Topotecan	Safety, Efficacy	Pending	Pending	Recruiting
NCT05652855	I/II	DB-1311	Anti-B7-H3 ADC	Advanced Solid Tumours (incl. SCLC)	DB-1311 Monotherapy	Safety, ORR	Pending	Pending	Not yet recruiting
NCT06801834	I	MHB088C	Anti-B7-H3 ADC	Advanced Solid Tumours	MHB088C Monotherapy	Safety, Tolerability	Pending	Pending	Recruiting
NCT06612151	I	YL201	Anti-B7-H3 ADC	SCLC	YL201 + Topotecan	Safety, Tolerability	Pending	Pending	Recruiting
TROP2									
(Ongoing trial)	III	Sacituzumab Govitecan	Anti-TROP2 ADC	Relapsed SCLC	SG vs. Topotecan/Amrubicin	PFS, OS	Pending	Pending	Recruiting
NCT05154604	I	SHR-A1921	Anti-TROP2 ADC	Advanced Solid Tumours (incl. SCLC)	SHR-A1921 Monotherapy	Safety, Tolerability	Pending	Pending	Unknown
SEZ6									
NCT05154604	I	ABBV-706	Anti-SEZ6 ADC	Advanced Solid Tumours (incl. SCLC)	ABBV-706 ± Chemo ± Budigalimab	Safety, Tolerability	Pending	Pending	Unknown

#### 3.1.1. Rovalpituzumab Tesirine (Rova-T)

Rova-T is the first ADC targeting DLL-3 [36], which delivers the cytotoxic payload Tesirine to induce DNA damage and thereby inhibit the proliferation of tumour cells overexpressing this receptor. A Phase I clinical trial [37] enrolled 74 small cell lung cancer patients, demonstrating an objective response rate (ORR) of 31%, a clinical benefit rate of 85%, a median progression-free survival (PFS) of 4.6 months, and a median OS of 5.8 months. These data indicate encouraging single-agent antitumour activity and manageable safety for Rova-T. Regrettably, subsequent studies failed to meet expectations. Based on the results of the Phase I study, the Phase II trial enrolled 339 subjects. All subjects received at least one cycle of Rova-T treatment. Primary endpoints included ORR, complete response (CR), OS, and PFS. Results demonstrated an ORR of 12.4% across all patients, 14.3% in DLL3-high expressors (≥75%), and 13.2% in DLL3-positive patients (≥25%). OS was 5.6 months for all patients, 5.7 months for those with high DLL3 expression, and 5.8 months for DLL3-positive patients. The impact of varying DLL3 expression levels on Rova-T efficacy remains unclear. Notable adverse events (AEs) included fatigue, pleural effusion, thrombocytopenia, and skin reactions, with a 63% incidence of grade 3–5 AEs. Phase II clinical trial results demonstrated that Rova-T did not significantly improve OS in patients with recurrent SCLC, failing to achieve the anticipated therapeutic effect [38]. The subsequent Phase III clinical trial was also rather disappointing. The TAHOE trial demonstrated that in patients with small cell lung cancer, Rova-T exhibited inferior OS compared to the current standard second-line chemotherapy agent topotecan, alongside a higher incidence of adverse reactions including pleural effusion, photosensitivity, and peripheral oedema [39]. In the MERU trial for extensive-stage small cell lung cancer (ED-SCLC), patients were prematurely terminated due to the Rova-T group failing to achieve a survival benefit. Furthermore, the incidence of grade 3 or higher drug-related toxicity was higher in the Rova-T group than in the placebo group [40]. Clinical trials combining Rova-T with immunotherapy [41] or platinum-based chemotherapy [42] also failed to demonstrate significant survival advantages. Ultimately, the drug’s clinical development programme was terminated, marking it a failure. While Rova-T’s remarkable early clinical efficacy had ignited considerable hope for SCLC treatment, it ultimately concluded in failure. Nevertheless, the discontinuation of this drug does not signify the failure of all DLL3-targeted therapies. The lessons learned remain highly relevant for numerous ongoing clinical programmes currently in development. The development trajectory of Rova-T highlighted that target expression alone was insufficient to guarantee ADC success and underlined the need for optimised linker–payload combinations and improved patient-stratification strategies beyond simple DLL3 positivity.

#### 3.1.2. SC-002

SC-002 is another ADC drug that targets DLL3 and is distinguished from Rova-T by a different chemical linker structure. Its early clinical study (NCT02500914) included patients with refractory SCLC/large cell neuroendocrine carcinoma (*n* = 35) treated with SC-002 and showed an ORR of 14% (5/35) and an ORR of 11.8% in patients screened by immunohistochemistry for DLL3 expression. In total, there was 66% adverse reactions in G3 and G4 and fatal TRAE in 1 (3%) patient. The most common adverse reactions included plasma cavity effusion, dyspnoea and, decreased platelets. Due to limited therapeutic efficacy and the occurrence of relatively severe adverse reactions, the study was discontinued [43]. The failure of SC-002 further emphasised the class-specific challenges of DLL3-targeting ADCs, notably on-target, off-tumour toxicities, and required innovative engineering to widen the therapeutic window.

#### 3.1.3. ZL-1310

ZL-1310 is an innovative ADC that targets DLL3 and is connected to a payload of topoisomerase I inhibitors using a cleavable linker [44]. A Phase I study (NCT06179069) is ongoing in patients with relapsed/refractory extensive-stage small cell lung cancer (r/r ES-SCLC) who have progressed after at least one prior platinum-based regimen. This study evaluates safety, ORR, duration of response (DOR), disease control rate (DCR), and pharmacokinetics. The study comprised a dose-escalation phase (Part 1A) and a randomised dose-optimisation/expansion phase (Part 2). Part 1A enrolled 28 patients receiving doses ranging from 0.8 to 2.8 mg/kg, with a median study duration of 5.1 months. Treatment-related adverse events (TRAEs) occurred in 89% of patients, with grade ≥3 TRAEs occurring in 39%. One patient experienced dose-limiting toxicity (neutropenia and thrombocytopenia). Five patients had dose reductions due to TRAEs, and five patients discontinued treatment. Common ≥ grade 3 TRAEs included anaemia (6 patients), neutropenia (5 patients), and thrombocytopenia (3 patients) [45]. Notably, among patients with brain metastases, the ORR was 80% and DCR reached 100% following treatment. ZL-1310 has demonstrated manageable safety and favourable antitumor activity in patients with refractory/relapsed advanced small cell lung cancer who have received prior treatments, with particularly significant efficacy in patients with brain metastases. This provides a novel weapon for controlling life-threatening brain metastases.

#### 3.1.4. FZ-AD005

FZ-AD005 is an ADC targeting DLL3. It specifically binds to DLL3 on the cell surface to form an antigen–antibody complex. Mediated by the antigen, endocytosed into cells, transported to lysosomes, and released as the active small-molecule drug DXd under the action of cathepsins. This subsequently inhibits topoisomerase I, inducing double-strand DNA breaks in target cells, arresting the cell cycle, and inducing apoptosis. This drug has demonstrated significant antitumour activity in both cellular and animal studies, with manageable toxicity and acceptable safety [46]. A first-in-human, open-label Phase I study (NCT06424665) evaluating advanced solid tumours is currently underway. This study aims to assess its safety, tolerability, pharmacokinetic profile, and preliminary antitumour activity. Primary endpoints include dose-limiting toxicities (DLTs), maximum tolerated dose (MTD), objective response rate (ORR), and adverse events (AEs). Secondary endpoints encompass PFS, OS, DOR, and maximum plasma concentration (Cmax) [47]. FZ-AD005 exemplified ongoing innovation in the DLL3-targeted ADC field, incorporating a topoisomerase I inhibitor payload to address limitations of earlier PBD based agents and to broaden the clinical utility of this target class.

#### 3.1.5. SHR-4849

In its first-in-human Phase I clinical trial, SHR-4849, a new ADC that targets DLL3, showed good antitumor activity and a controllable safety profile in patients with recurrent small cell lung cancer(r-SCLC) who had previously undergone conventional therapy. Patients with metastatic or recurrent small cell lung cancer whose disease has advanced after receiving standard treatment were recruited for this study. Every three weeks, SHR-4849 was injected intravenously. During the dose-escalation phase, multiple dose levels ranging from 0.8 mg/kg to 5.0 mg/kg were evaluated, with 2.4, 3.0, and 3.5 mg/kg ultimately selected as the expansion cohort doses. Significant antitumour efficacy was demonstrated in patients receiving doses ≥2.4 mg/kg. Among all evaluable patients, the ORR reached 73.2%, with a DCR as high as 93.0%. Notably, the DCR achieved 97.1% in the second-line treatment subgroup and 100% in patients with brain metastases. The safety profile of SHR-4849 was manageable, with overall good tolerability. These preliminary findings support the continued advancement of SHR-4849’s clinical development as a potential treatment option for patients with recurrent small cell lung cancer [48].

### 3.2. B7H3-Targeting ADCs

B7-H3, as a member of the B7 immunoregulatory family, exhibits low expression in normal tissues but demonstrates moderate to high expression in approximately 65% of SCLC. This expression correlates with greater tumour burden, enhanced metastatic potential, and poorer prognosis [49]. SCLC exhibits relatively low T-cell and B-cell infiltration, with high levels of B7-H3 expression potentially mediating immune evasion in SCLC. Consequently, B7-H3 has emerged as a key therapeutic target in SCLC treatment.

#### 3.2.1. DS-7300a

DS-7300a is an ADC that targets B7-H3. Topoisomerase I inhibitor (I-DXd) is attached to a humanised anti-B7-H3 monoclonal antibody via a cleavable tetrapeptide linker. The compound has demonstrated favourable antitumour efficacy in both in vitro and in vivo studies [50]. The IDeate-Lung01 trial (NCT05280470) was a multicenter, open-label, Phase II study evaluating I-DXd in previously treated extensive-stage SCLC (ES-SCLC) patients. A recommended dose of 12 mg/kg every three weeks was established in the initial phase and further assessed in an expansion cohort (*n* = 137). Patient characteristics included a median age of 63 years; 48.9% were Asian, 38.0% had brain metastases, and 40.1% had liver metastases. Most (76.6%) had received ≥2 prior lines of therapy, including immune checkpoint inhibitors (81.0%), topoisomerase I inhibitors (32.1%), and DLL3-targeted agents (8.0%).

As of March 3, 2025, with a median follow-up of 12.8 months, I-DXd exhibited robust and durable antitumor activity. The blinded independent central review (BICR)-assessed ORR was 48.2%, with a DCR of 87.6% and median DOR of 5.3 months. Second-line patients achieved an ORR of 56.3% and DCR of 96.9%. Median PFS and OS were 4.9 and 10.3 months overall, and 5.6 and 12.0 months in the second-line subgroup, respectively. Notably, in patients with baseline brain metastases (*n* = 65), the intracranial ORR was 46.2% [51]. Therefore, on 18 August 2025, DS-7300a received breakthrough therapy designation from the US Food and Drug Administration (FDA) for the treatment of adult patients with E-SCLC that has progressed following platinum-based chemotherapy [52]. This marks a breakthrough advancement in the field of ADCs for SCLC. The profound efficacy of I-DXd, including its notable intracranial activity, solidifies B7-H3 as a leading target in SCLC and validates the Deruxtecan platform as a transformative technology for ADCs beyond their original indications.

#### 3.2.2. HS-20093

HS-20093 is a B7-H3–directed ADC that has shown antitumor activity in patients with advanced solid tumours (NCT05276609). This study enrolled patients with ES-SCLC who had previously received platinum-based chemotherapy and no more than three lines of systemic therapy, the median number of prior systemic regimens was 2. Among the participants, 73.2% had received prior immunotherapy, and 14.3% had baseline brain metastases. Patients were administered HS-20093 at either 8.0 mg/kg Q3W (*n* = 31) or 10.0 mg/kg Q3W (*n* = 25). The primary endpoint was objective response rate (ORR). Among the 53 evaluable patients, the ORR was 61.3% in the 8.0 mg/kg cohort and 50.0% in the 10.0 mg/kg cohort. The median DOR was 6.4 months and 8.9 months, respectively, and median PFS was 5.9 months and 7.3 months, respectively. The most common adverse events consisted of gastrointestinal and hematologic toxicities, including neutropenia (39.3%), leukopenia (33.9%), lymphopenia (25.0%), thrombocytopenia (17.9%), and anaemia (16.1%) [53]. The study’s limitations relate chiefly to its early stage of clinical development, the limited representativeness of the target population and the occurrence of haematological toxicities. The promising activity of HS-20093 underscores the competitive landscape for B7-H3 targeting, yet its haematological toxicity profile emphasised the critical need to refine the therapeutic index of next-generation ADCs in this class.

#### 3.2.3. MHB088C (QLC5508)

MHB088C is an ADC targeting B7-H3, it’s potent SuperTopoi payload exhibits cytotoxic activity 5 to 10 times greater than Dxd. Preliminary results from an ongoing Phase I/II clinical trial indicate that MHB088C is generally well tolerated and shows early signs of clinical activity. This study focused on analysing efficacy and safety outcomes in the recurrent ES-SCLC patient subgroup. The investigation comprised two phases: dose escalation (Part 1) and dose expansion (Part 2). Part 1 evaluated safety and tolerability across a 0.8 to 4.0 mg/kg dose range administered every two weeks (Q2W) or every three weeks (Q3W). Based on these findings, Part 2 selected three dosing regimens (1.6 mg/kg Q2W, 2.0 mg/kg Q2W, and 2.4 mg/kg Q3W) to further evaluate the antitumour activity and safety of MHB088C in specific tumour types, including small cell lung cancer.

As of 3 January 2025, 91 patients with recurrent ES-SCLC had received at least one dose of MHB088C, allocated to three cohorts: 1.6 mg/kg Q2W (*n* = 28), 2.0 mg/kg Q2W (*n* = 33) and 2.4 mg/kg Q3W (*n* = 30). In this heavily pretreated population, MHB088C demonstrated encouraging antitumour activity, with objective response rates (ORR) of 42.9%, 57.6%, and 46.7% across the three cohorts, respectively. Median PFS was 5.5 months, 5.9 months, and 5.5 months, respectively. Regarding safety, MHB088C demonstrated overall manageable toxicity. The most common ≥grade 3 treatment-related adverse events were neutropenia, thrombocytopenia, and anaemia. The 1.6 mg/kg and 2.0 mg/kg Q2W regimens exhibited superior haematological safety, with incidence rates below 10% for these events. Only one case (1.0%) of mild interstitial lung disease (ILD) was reported.

In summary, MHB088C demonstrated promising clinical efficacy and acceptable safety in previously treated patients with advanced small cell lung cancer. Based on these findings, a Phase III clinical trial was planned to further compare the efficacy of MHB088C versus standard chemotherapy in patients with recurrent advanced small cell lung cancer [54].The development of MHB088C, featuring a highly potent novel payload, represented a significant advance in ADC payload technology and demonstrated that increased cytotoxicity could be balanced with acceptable safety through optimised dosing schedules.

### 3.3. Seizure-Related Homologue 6 (SEZ6) -Targeting ADCs

SEZ6 is a type I transmembrane protein involved in neuronal development. SEZ6 is predominantly expressed in SCLC and high-grade neuroendocrine tumours, whilst exhibiting low expression in normal cells. Over 80% of SCLC cases express SEZ6, demonstrating its potential as a therapeutic target in SCLC treatment [55].

#### 3.3.1. ABBV-011

ABBV-011 is an ADC comprising a monoclonal antibody targeting the SEZ6 antigen, conjugated via a non-cleavable linker to the cytotoxic drug kanamycin. Although ABBV-011 demonstrated efficacy in preclinical studies and patient-derived xenograft (PDX) models of SCLC [56], subsequent research failed to achieve the desired therapeutic outcomes. The first Phase I study evaluating ABBV-011 in patients with recurrent SCLC (NCT03639194) enrolled 99 subjects who received ABBV-011 monotherapy (1.0 mg/kg every three weeks). Results indicated an objective response rate (ORR) of 19% in the evaluable population (*n* = 98). Within the 1 mg/kg dose-expansion cohort (*n* = 60), ORR increased to 25% with a median PFS of 3.5 months, suggesting a potential dose–response relationship. Regarding drug safety, fatigue, nausea, and thrombocytopenia were the most common adverse events. Treatment-emergent adverse events (TEAEs) related to liver function occurred in 42% of the overall population, with 12% being grade ≥3 events, primarily manifesting as hyperbilirubinaemia and elevated transaminases [57]. Given its significant hepatotoxicity in humans, the ABBV-011 drug study has now been discontinued.

#### 3.3.2. ABBV-706

ABBV-706 is an SEZ6-targeted ADC drug whose payload is a topoisomerase I inhibitor (TOP1i) linked via a Valine-alanine spacer. ABBV-706 recognises and internalises tumour cells expressing SEZ6, enabling precise delivery of the tumour-killing payload. In a Phase I study (NCT599984) of 80 patients with recurrent/refractory small cell lung cancer (R/R SCLC), subjects were randomised to receive either 1.8 mg/kg (*n* = 41) or 2.5 mg/kg (*n* = 39) every three weeks. The study aimed to evaluate efficacy, safety, pharmacokinetics, and determine the recommended Phase II dose (RP2D). Results showed comparable overall response rates (ORR) between both dose groups in the overall population, but the 1.8 mg/kg group demonstrated superior DOR and PFS. Efficacy was more pronounced in Top1i-naïve and/or second-line treatment patients, with ORRs of 62.1% and 81.3% in the 1.8 mg/kg group, respectively. The 1.8 mg/kg group demonstrated superior safety, with a grade ≥ 3 treatment-related adverse event (TRAE) incidence of 49%. Common TRAEs included anaemia and neutropenia. Overall TRAE-related discontinuation, interruption, and dose reduction rates were 9%, 44%, and 28%, respectively. ABBV-706 demonstrated manageable safety and favourable antitumor activity in R/R SCLC. Notably, the objective response rate (ORR) in patients with brain metastases reached 62.5% in the 1.8 mg/kg dose group [58]. Based on these findings, a Phase II clinical trial has been initiated to further validate its efficacy. ABBV-706 is a more successful ADC developed following ABBV-011. The pronounced efficacy of ABBV-706, particularly in brain metastases, has revitalised the SEZ6 target class and shows that strategic optimisation of payload and linker can surmount the limitations of predecessor ADCs, thus paving the way for a new generation of neuroendocrine cancer therapeutics. We believe it will continue to demonstrate efficacy in subsequent clinical trials; however, its higher incidence of adverse reactions necessitates further validation through Phase II and III clinical trials.

### 3.4. CD56-Targeting ADCs

CD56 positive expression rates can be over 90% in SCLC with neuroendocrine features [59]. Given that CD56 serves as a key diagnostic marker for identifying neuroendocrine-derived cells (including SCLC) in immunohistochemistry, its specific expression on the cell surface also renders it a potential therapeutic target for treating this aggressive cancer.

#### Lorvotuzumab Mertansine (LMGN901, LM)

LM is a complex formed by conjugating the CD56 antibody N901 with the microtubule-like inhibitor DM1, and it is a neurocell adhesion molecule [60]. Following binding to CD56 on the surface of target cells, LM enters the cell via internalisation. Subsequently, the linker is cleaved, releasing DM1, which induces cell death by inhibiting tubulin polymerisation [61]. LM shows potent antitumor activity in mice against a xenograft model of SCLC [62]. In a Phase 1/2 study, LM combined with chemotherapy (carboplatin + etoposide) showed a slight improvement in ORR compared to chemotherapy alone (67.1% vs. 59.0%), but PFS was not prolonged (6.2 vs. 6.7 months); 18 of 94 patients treated with LM died due to treatment-related adverse events (TRAE). This also indicates that although CD56 is a noteworthy target in small cell lung cancer, further optimisation of treatment regimens requires reducing toxicity and enhancing efficacy [63].

### 3.5. TROP-2-Targeting ADCs

Trophoblast Surface Antigen 2 (TROP2) is a member of the tumour-associated calcium signal transduction protein family, overexpressed in numerous solid tumours and closely associated with tumour proliferation, invasion, and poor prognosis. TROP2 exhibits low expression in normal tissues, high expression in tumour cells, and rapid internalisation properties [64]. These characteristics indicate TROP2 possesses favourable safety and strong targeting potential, thereby qualifying it as an effective target for ADCs. In the treatment of SCLC, ADC drugs targeting TROP2 have also demonstrated their unique appeal [55].

#### 3.5.1. Sacituzumab Govitecan (SG)

SG comprises the TROP-2 monoclonal antibody hRS7 linked to the active metabolite of irinotecan (SN-38) via the cleavable linker CL2A [65,66]. In a Phase 1/2 IMMU-132-01 basket trial, the ORR of SG for the treatment of patients with recurrent SCLC was 17.7%, with mDOR and mOS of 5.7 months and 7.1 months, respectively [67,68]. Another Phase II, multi-cohort, open-label basket study, TROPiCS-03 (NCT03964727), aims to evaluate the efficacy and safety of SG as a second-line treatment regimen in patients with ES-SCLC who have previously received platinum-based chemotherapy and anti-PD-(L)1 immunotherapy [69]. Enrolled patients received an intravenous infusion of SG (10 mg/kg) on Days 1 and 8 of a 21-day treatment cycle. The primary endpoint was objective response rate (ORR); key secondary endpoints included DOR, PFS, OS and safety. Certain efficacy endpoints were assessed both by investigators and by blinded independent central review (BICR). The study enrolled 43 patients with a median follow-up duration of 12.3 months. Results demonstrated an investigator-assessed ORR of 41.9%, with 18 partial responses. Median DOR was 4.73 months, median PFS was 4.40 months, and median OS was 13.60 months, with BICR assessments largely consistent with investigator evaluations. Subgroup analysis indicated that SG induced tumour response in both platinum-sensitive (*n* = 23) and platinum-resistant (*n* = 20) patients, with ORRs of 47.8% and 35.0%, respectively. Regarding safety, 74.4% of patients reported ≥grade 3 treatment-related adverse events (TEAEs). No patients discontinued treatment because of TEAEs, although one death related to neutropenic sepsis was reported. These findings confirm that SG as a second-line therapy delivered clinically meaningful antitumour activity in pretreated ES-SCLC patients, irrespective of platinum sensitivity status. This aligns with data reported for SG in triple-negative breast cancer and urothelial carcinoma and further indicates its favourable tolerability. The results also reaffirm the therapeutic potential of ADCs in ES-SCLC. However, as this study remained a Phase II trial, its true efficacy and safety profile clearly required further validation through Phase III research.

#### 3.5.2. SHR-A1921

SHR-A1921 is a novel ADC comprising a humanised anti-TROP-2 monoclonal antibody, a tetrapeptide cleavable linker, and a topoisomerase I inhibitor [70]. In a Phase I human trial (NCT05154604) featuring an expanded cohort for SCLC, patients with extensive-stage SCLC who progressed following platinum-based chemotherapy were enrolled. Regardless of TROP-2 expression status, all received SHR-A1921 at a dose of 3.0 mg/kg every three weeks. As of 19 February 2024, 17 patients had been enrolled: 52.9% had received ≥2 prior lines of therapy, 64.7% had prior anti-PD-1/PD-L1 therapy, with a median follow-up of 5.3 months. Results showed an objective response rate of 33.3% and a disease control rate of 66.7% among 15 evaluable patients. Median PFS was 3.8 months, with median OS not yet reached. All patients experienced treatment-related adverse events, most commonly stomatitis (58.8%), nausea (35.3%), and vomiting (23.5%). Grade 3 or higher adverse events occurred in 35.3% of patients, with stomatitis (11.8%) being the most frequent. No treatment discontinuations due to treatment-related adverse events were observed [71]. Collectively, these findings position SHR-A1921 as a compelling candidate warranting further validation in later-phase trials, highlighting the ongoing evolution in ADC engineering to maximise clinical benefit in SCLC.

## 4. Small-Molecule Drug Conjugates (SMDCs): New Options for Targeted Therapy Based on ADCs

Building on ADC design principles, the development of lightweight targeted therapeutics such as SMDCs has emerged as a novel strategy in targeted cancer therapy. SMDCs consist of three components: a small-molecule targeting ligand, a cytotoxic payload and a cleavable linker. Compared with ADCs, SMDCs penetrated and distributed more uniformly within tumour tissues because of their small-molecule character. They also offered distinct advantages, including lower production costs and lack of immunogenicity, and demonstrated broad clinical application prospects. This approach may overcome the limitations of ADCs and open entirely new avenues for ADC research [72].

### 4.1. PEN-221

PEN-221 targets the SSTR2 protein, with the cytotoxic moiety being the medenin derivative DM-1. SSTR2 is overexpressed in SCLC while its expression in normal tissues is limited [73,74]. In vivo studies demonstrate that PEN-221 precisely targets tumours overexpressing SSTR2, inducing profound and sustained tumour regression across multiple xenograft mouse models. The compound exhibits excellent tolerability in experimental animals, confirming its substantial potential as a targeted therapy. Clinical trial data further corroborates the drug’s efficacy and safety profile. Data presented at the 2018 ASCO meeting showed disease stabilisation of up to 12 weeks in patients with aggressive, advanced, SSTR2-positive small cell lung cancer treated with PEN-221 [75]. These data were considered a major breakthrough in the treatment of small cell lung cancer, and the SMDC drug has generated greater interest as a result.

### 4.2. PEN-866

PEN-866 has a targetable binding to activated heat shock protein (HSP90) at one end and an anti-cancer payload, SN-38, at the other. HSP90 is a molecular chaperone protein that is highly activated in the hostile tumour environment of various solid tumours, but remains relatively dormant in normal tissues and was once considered a potential target for oncology drug development [76]. PEN-866 demonstrates an extended plasma half-life in patients with advanced solid tumours and exhibits favourable stability of its SN-38 payload during limited plasma exposure. Results of a Phase I clinical trial enrolling 21 patients from seven cohorts showed that PET-866 was well tolerated in patients with advanced solid tumours, with the expected adverse event profile and antitumour activity [77]. PEN-866 is currently in a Phase II solid tumour basket trial in the U.S. The approval of the drug for clinical trials in SCLC and NSCLC in China in 2022 means that the drug is also about to enter clinical development in China.

## 5. Challenges Facing ADC Drugs and Resolution Strategies

### 5.1. ADCs Drug Toxicity: A Critical Factor That Cannot Be Overlooked

**Figure 4:**
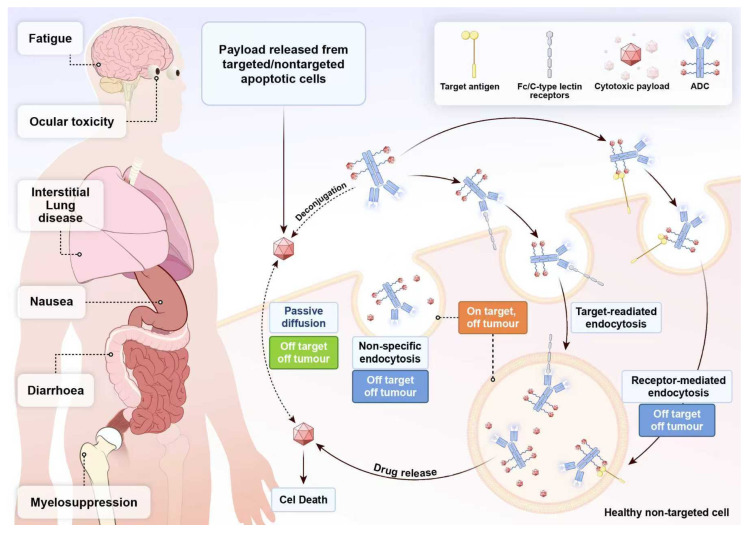
Mechanism of action and key adverse events of ADCs. ADC drugs are designed to selectively deliver cytotoxic payloads to tumour cells. The process begins when the ADC antibody component binds to its target antigen on the tumour cell surface, leading to (1) target-mediated endocytosis. The ADC-antigen complex is internalised and trafficked to endosomes/lysosomes, where the linker is cleaved, resulting in drug release and subsequent cell death. In addition, several off-target pathways contribute to systemic toxicity: (2) Receptor-mediated endocytosis in healthy, off-target cells expressing the receptor (e.g., FcγR). (3) Non-specific endocytosis in various cell types. (4) Passive diffusion of the payload after premature linker cleavage in circulation. ADC-mediated off-target effects lead to payload release in healthy non-targeted cells, causing organ damage and common adverse events such as interstitial lung disease, myelosuppression, nausea, diarrhoea, fatigue, and ocular toxicity.

Although ADCs demonstrate potent antitumour efficacy in small cell lung cancer, their associated adverse reactions remain a significant concern that cannot be overlooked (Figure 4). A meta-analysis encompassing 138 ADC clinical trials indicated that the majority of AEs were grade 1–2 in severity, whilst the incidence of grade ≥3 AEs stood at 6.2% [78]. Another meta-analysis involving 169 clinical trials and 22,492 patients similarly demonstrated that treatment-related adverse events of all grades occurred in 91.2% of patients receiving ADC therapy, with grade 3 or higher events occurring in 46.1%. Among these, 153 studies reported fatal adverse events associated with ADC therapy, with an overall mortality rate of 1.3%. The most common direct causes of death were pneumonia, sepsis and respiratory failure [79]. Furthermore, compared with conventional therapies, ADC treatment significantly increased the incidence of infectious adverse events, including upper respiratory tract and urinary tract infections. This greater susceptibility to infection may have resulted from ADCs’ unintended effects on non-malignant cells that express the target antigen, thereby compromising immune function [80]. These adverse effects primarily stem from the toxic effects of ADCs on normal human cells, with mechanisms categorised into two main types: off-target toxicity and on-target toxicity [81]. Off-target toxicity refers to drug toxicity occurring in cells that do not express the target antigen, representing the most critical determinant of ADC toxicity. Specifically, the target selection for ADCs is typically based on proteins specifically expressed on the surface of tumour cells. However, expression of these antigens in non-tumour tissues may lead to the erroneous delivery of cytotoxic drugs to non-tumour cells, thereby inducing off-target toxicity. Additionally, linker instability or specific factors may cause premature release of the payload in body fluids, non-tumour tissues, or the tumour microenvironment before reaching target cells, similarly triggering off-target toxicity. On-target toxicity refers to toxicity arising from ADC binding to normal cells expressing the target antigen. This occurs because certain non-tumour cells possess receptors capable of binding to the Fc fragment of the IgG antibody within the ADC, thereby mediating uptake of the ADC drug by non-tumour cells [82]. Furthermore, non-specific endocytic mechanisms (such as macropinocytosis and micropinocytosis) may also result in the uptake of the entire ADC or its free payload by normal cells, subsequently generating toxicity [81].

The toxicity issues with ADCs in clinical practice mostly show up as treatment-related hazards, such as organ damage mediated by particular targets, hepatotoxicity, ocular toxicity, and bone marrow suppression. However, effective intervention against potential toxicities is made possible by systematic risk management strategies, such as the early identification of high-risk populations, the establishment of toxicity early warning indicators, the development of tiered management protocols, and the implementation of comprehensive risk monitoring plans from the very beginning of clinical development (e.g., first-in-human trials). Additionally, by modifying pre-treatment protocols, optimising dose intervals, or adjusting dosing regimens, clinical tolerability can be greatly increased for some anticipated adverse events without sacrificing efficacy. Therefore, by thorough, nuanced risk management, ADCs’ overall safety profile can be controlled even with their intrinsic toxicological load. In drug design, bispecific ADCs may be selected, employing multidimensional synergistic strategies to significantly enhance therapeutic safety. This ensures the drug specifically accumulates and internalises only within tumour cells co-expressing both targets, thereby effectively circumventing toxicity to single-target-positive healthy tissues. Secondly, in linker design, employing highly stable chemical structures reduces premature payload release in plasma, whilst introducing hydrophilic groups enhances ADC molecular hydrophilicity, effectively diminishing non-specific binding to normal tissues. Additionally, simultaneous administration of Fab fragments that can bind the payload specifically during ADC delivery can actively neutralise any unbound toxins that may be in the bloodstream, thus better managing the risks of systemic exposure [83].

### 5.2. ADCs Drug Resistance: The Looming Threat in Targeted Therapy

**Figure 5:**
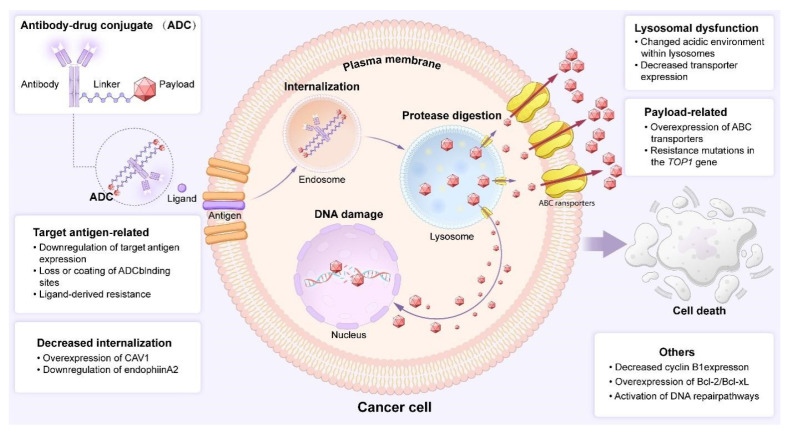
Mechanisms of resistance to ADCs. Tumour cells evade ADC cytotoxicity through sequential resistance mechanisms: (1) impaired target recognition, via antigen downregulation, epitope masking, or soluble ligand competition; (2) disrupted internalization and trafficking, due to altered endocytic pathways; (3) failed payload release, resulting from lysosomal dysfunction; (4) enhanced payload efflux, mediated by ABC transporter upregulation (e.g., ABCB1); and (5) altered payload targets and survival pathways, involving target downregulation, anti-apoptotic adaptation, or enhanced DNA repair. The convergence of these pathways underscores the multifactorial nature of ADC resistance.

Despite the expanding therapeutic promise of ADCs in clinical practice, the emergence of drug resistance remains a significant and inevitable challenge (Figure 5). A pivotal factor underpinning this resistance across oncology therapeutics is tumour heterogeneity [84]. For ADCs precisely targeting surface antigens, their efficacy is highly dependent on the uniform expression of the antigen. Tumour cells can evade recognition and binding by downregulating or altering the expression of the target antigen. Furthermore, the development of ADC resistance is intrinsically linked to inefficiencies in its multi-step mechanism of action [85]. Any disruption in the cascade—from the internalisation of the antigen–antibody complex and lysosomal trafficking to the efficient release and subsequent intracellular retention of the cytotoxic payload—can confer resistance [86]. Unlike breast cancer, non-small cell lung cancer, and other malignancies, small cell lung cancer presents a more complex challenge regarding ADC resistance due to the absence of specific targetable structures and well-defined signalling pathways. Compounded by the lack of dedicated clinical research, the underlying mechanisms remain unclear to this day [87]. Consequently, there is an urgent need for dedicated clinical trials targeting the high recurrence and high heterogeneity of SCLC to elucidate its resistance mechanisms. This is not only crucial for guiding clinical practice but also key to achieving therapeutic breakthroughs. Subsequent research should focus on exploring novel targets for small cell lung cancer, optimising treatment regimens (including drug dosages and administration frequencies), and progressively overcoming the challenge of drug resistance. At the level of drug design technology, emphasis should be placed on enhancing monoclonal antibodies, payloads, linkers, and conjugation techniques to mitigate resistance and thereby improve therapeutic efficacy.

## 6. Future Development Direction of ADC

### 6.1. Synergy in Action: Reshaping the Therapeutic Landscape with ADC-Based Combinations

ADCs have undergone three generations of technological breakthroughs, with techniques such as site-specific conjugation and highly stable linkers significantly enhancing therapeutic efficacy. However, issues including drug toxicity and resistance continue to constrain the deepening of clinical outcomes [88]. Therefore, the combination of ADCs with other therapeutic modalities holds promise for reshaping the treatment landscape of multiple malignancies. Combination therapies involve the concurrent use of ADCs alongside chemotherapy, targeted therapies, and immunotherapy, marking an evolution in cancer treatment from ‘single-agent regimens’ to ‘synergistic strategies’. However, in the field of SCLC, combination therapies incorporating ADCs still face limitations. A Phase I clinical trial (NCT04826341) aimed to evaluate the safety and efficacy of SG in combination with the ATR inhibitor Berzosertib for the treatment of patients with advanced solid tumours. Results demonstrated tumour regressions in two patients with neuroendocrine prostate cancer and one patient with small cell lung cancer [89]. Although this demonstrates the efficacy of ADC combined with targeted therapy, it remains limited to individual cases at present. Regarding synergistic immunotherapy, the payload released by ADCs can induce immunogenic cell death (ICD) within tumour cells, thereby promoting the maturation and activation of antigen-presenting cells (APCs) and enhancing T-cell infiltration and function. Concurrently, anti-PD-1 antibodies block the PD-1/PD-L1 signalling pathway, thereby releasing T-cell suppression by tumours and further amplifying the immunotherapeutic efficacy of combination therapy. While these preliminary findings offer initial validation for ADC-immunotherapy combinations, their definitive efficacy must be confirmed through larger, controlled clinical trials [90]. Regarding the combination therapy of ADCs with chemotherapy, its efficacy has been demonstrated to some extent in breast cancer, non-small cell lung cancer, urothelial carcinoma and other cancer types, yet there remains a gap in the field of SCLC [91]. We firmly believe that in future research, ADC combination therapy holds great promise for SCLC.

### 6.2. Optimising ADC Therapeutics: Rational Design and Enhanced Safety Management

Another major development direction for ADCs lies in further optimisation at the drug design level. ADCs represent a class of structurally complex targeted therapeutics, where molecular design directly influences clinical efficacy and safety. In recent years, multiple technological innovations announced by the European Society for Medical Oncology (ESMO) are propelling ADCs towards higher precision and reduced toxicity, transforming them into more selective “magic bullets”. Technological evolution manifests across three core domains: firstly, the application of site-specific conjugation techniques enables precise control of the drug/antibody ratio (DAR), thereby enhancing pharmacokinetic stability and therapeutic consistency. For instance, ABBV-706, by optimising its DAR to 6, demonstrated sustained tumour regression in preclinical lung cancer models [92]. Secondly, the development of novel, highly efficient payloads has amplified antitumour activity and holds promise for overcoming resistance mechanisms. For instance, Sac-TMT, utilising a topoisomerase I inhibitor, demonstrated significant efficacy even in patients who had failed multiple prior treatments [93]. Thirdly, continuous optimisation of linkers improves system stability, reducing non-specific release of payloads into the bloodstream and thereby lowering off-target toxicity to normal tissues. Regarding safety management, the clinical safety profile of next-generation ADCs continues to improve. Taking Dato-DXd as an example, its incidence of grade 3 or higher treatment-related adverse events was only 21%, significantly lower than traditional chemotherapy, with a low incidence of neutropenia at 1%, indicating superior patient tolerability [94]. Furthermore, concerning the ILD risk specific to ADCs, a comprehensive management consensus has been established, encompassing high-risk patient identification, early imaging screening, and tiered intervention protocols. This strategy demonstrates significant efficacy in practice; during ABBV-400 treatment, the incidence of ILD across all grades was controlled at 4.8%, underscoring the critical role of systematic management in mitigating drug-related risks [95].

Researchers have also further expanded the payloads of ADCs. Immune-stimulating antibody–drug conjugates (ISACs) represent a novel class of targeted immunotherapeutic agents aimed at addressing the limitations of conventional tumour treatment strategies. Structurally similar to ADCs, ISACs consist of tumour-targeting monoclonal antibodies, chemical linkers, and immune-stimulating payloads, such as TLR 7/8 or STING agonists. In contrast to ADCs, which induce direct cytotoxicity in antigen-expressing tumour cells following internalisation, ISACs reprogram the tumour microenvironment (TME) through their payloads. By activating innate immune cells, including dendritic cells and macrophages, and enhancing antigen presentation, ISACs can convert immunologically ‘cold’ tumours into ‘hot’ tumours. This transformation initiates systemic antitumor immunity and may establish long-term immune memory, marking a significant transition from a paradigm of ‘direct killing’ to one focused on ‘activation of innate immunity.’ [96]. In the treatment of SCLC, ISAC may exert an immunostimulatory effect; its efficacy and safety remain to be further validated through clinical trials. Moreover, the development of bispecific ADCs is progressively reshaping the therapeutic landscape for SCLC. For instance, the bispecific ADC BL-B01D1, targeting EGFR/HER3, has demonstrated encouraging efficacy in SCLC treatment. BL-B01D1 is an EGFR × HER3 bispecific ADC with a topoisomerase I inhibitor payload. A Phase I clinical trial evaluated the efficacy and safety of BL-B01D1 in patients with locally advanced or metastatic SCLC. The median follow-up duration was 7.5 months. The efficacy analysis included 33 subjects, with an ORR of 39.4%. One patient achieved complete response (CR), twelve demonstrated partial response (PR), with PFS at 5.5 months and median OS at 13.1 months. Based on these encouraging results, a Phase III study (NCT06422988) of BL-B01D1 has commenced in ES-SCLC patients who progressed during or after platinum-based therapy [97].

## 7. Discussions

Small cell lung cancer, a refractory tumour with limited efficacy of previous drugs, has broken the silence in the field of SCLC treatment with the advent of immunological drugs and anti-angiogenic drugs in recent years, but its long-term survival has still not been significantly improved. The advent of ADCs represents not only a significant watershed in SCLC treatment but also marks precision oncology’s entry into a new phase guided by molecular subtyping. This cutting-edge strategy holds promise to reshape traditional SCLC treatment paradigms, ushering in a new era of therapy and offering renewed hope for numerous advanced SCLC patients resistant to immunotherapy and chemotherapy. T-DXd is the first ADC treatment for lung cancer and is the most advanced and well-developed ADC research for lung cancer [98,99]. However, the development of ADCs in SCLC has not been without setbacks. The failure of early programmes such as Rova-T and ABBV-011 serves as a reminder that despite highly specific expression of targets like DLL3 in SCLC, their biological functions and regulatory networks remain profoundly complex. Meanwhile, cytotoxic-related adverse reactions and drug resistance remain the primary challenges facing current ADC therapies. Nevertheless, next-generation ADCs such as ZL-1310 and ABBV-076, demonstrate potential therapeutic value, offering renewed hope for patients with drug-resistant or refractory disease. To expand the therapeutic window, there is a need to explore optimal ADC design strategies in new drug development, including the selection of targets and payloads, and the optimisation of coupling technologies to achieve both efficacy and safety; for example, bispecific ADCs can recognise two targets or two epitopes of one target, helping to reduce off-target rates and increase the toxicity of killing tumour cells [100]. In addition, there is a need to further search for biomarkers that can predict the prognosis of ADC treatment, as well as to explore the mechanisms of ADC resistance and strategies to reverse it. The combination of ADCs with immunotherapy enhances tumour suppression and overcomes resistance. We anticipate that ongoing breakthroughs in ADC research will significantly improve treatment outcomes for patients with small cell lung cancer and other neuroendocrine tumours.

Search strategy and methods: To ensure a comprehensive and systematic review of ADCs in SCLC, a rigorous search of the literature was conducted across multiple electronic databases, including PubMed, Web of Science and ClinicalTrials.gov. The search strategy aimed to capture all relevant preclinical, clinical and translational studies published or registered between January 2000 and October 2025. The following key terms and their combinations were used: “antibody-drug conjugate,” “ADC,” “small cell lung cancer,” “SCLC,” “DLL3,” “B7-H3,” “TROP2,” “SEZ6,” “CD56,” “Rovalpituzumab Tesirine,” “DS-7300,” “Ifinatamab Deruxtecan,” “Sacituzumab Govitecan,” “clinical trial,” and “mechanism.” Inclusion criteria were: original research articles, clinical trials (any phase), meta-analyses and authoritative reviews; studies addressing ADC structure, mechanism, efficacy, safety or resistance in SCLC; and publications in English or with an English abstract. Exclusion criteria were: non-ADC therapies; studies not specific to SCLC; case reports, unless no larger trials were available; and publications without peer review or without an available full text. The screening process comprised an initial title and abstract review followed by a full-text assessment for eligibility. Data were systematically extracted from eligible studies and included study design, patient characteristics, intervention, efficacy endpoints (for example ORR, PFS, OS), and safety outcomes. Ongoing or recently completed clinical trials were monitored via ClinicalTrials.gov to capture the latest developmental updates and interim results. All references were managed and deduplicated using EndNote X9.

## 8. Conclusions 

In this review, we synthesise the current evidence supporting the evolving role of ADCs in SCLC. ADCs represent a promising class of therapeutics for SCLC, driven by the selective overexpression of target antigens such as DLL3, B7-H3, TROP-2 and SEZ6 in tumour tissue, which provides a clear rationale for their use. Evidence from preclinical and clinical studies indicates that ADCs exert antitumour effects via targeted cytotoxic delivery, enabling precise eradication of malignant cells while sparing normal tissue. Although early-generation ADCs, including Rova-T and ABBV-011, were discontinued because of limited efficacy or unacceptable toxicity, next-generation constructs such as ZL-1310, ABBV-706, and DS-7300a demonstrated improved therapeutic profiles and clinical potential. These advances underscore the growing importance of ADC optimisation in SCLC therapy and emphasise the effects of target selection, linker stability and payload potency on treatment outcomes. Importantly, accumulating clinical data have supported the efficacy of ADCs in patients with relapsed or refractory SCLC, a group with historically poor prognosis and few options. However, several challenges must be addressed systematically to realise the full therapeutic potential of ADCs. These include mitigating both target-specific and off-target toxicities, elucidating and overcoming resistance mechanisms such as antigen downregulation, impaired internalisation and payload efflux, and identifying robust predictive biomarkers for patient stratification. Notably, although a growing body of clinical data have confirmed the favourable efficacy of ADCs in patients with recurrent or refractory small cell lung cancer, several challenges must be systematically addressed to fully realise their therapeutic potential. These challenges include mitigating target specificity and off-target toxicity and elucidating and overcoming resistance mechanisms such as antigen downregulation, internalisation barriers and drug efflux. Future research should prioritise the development of bispecific ADCs and tissue-specific targeting strategies and should explore combination regimens with agents such as immune checkpoint inhibitors.

## Data Availability

All the data used in this study had been publicly available.

## Abbreviations

ADC	Antibody–drug conjugates
AEs	Adverse Events
BICR	Blinded Independent Central Review
Cmax	Maximum Plasma Concentration
CR	Complete Response
DCR	Disease Control Rate
DLTs	Dose-Limiting Toxicities
DOR	Duration of Response
ED	Extensive Disease
ESMO	European Society for Medical Oncology
FDA	U.S. Food and Drug Administration
ICI	Immune Checkpoint Inhibitors
ILD	Interstitial Lung Disease
LM	Lorvotuzumab Mertansine
mOS	Median overall survival
MTD	Maximum Tolerated Dose
NSCLC	Non-Small Cell Lung Cancer
ORR	Objective Response Rate
OS	Overall Survival
PDX	Patient-derived xenograft
PFS	Progression-Free Survival
RP2D	Recommended Phase II Dose
SCLC	Small Cell Lung Cancer
SEZ6	Seizure-related homologue 6
SG	Sacituzumab Govitecan
SMDC	Small-Molecule Drug Conjugates
STTR2	Somatostatin Receptor 2
TEAEs	Treatment-Emergent Adverse Events
TROP-2	Trophoblast Cell Surface Antigen 2
